# Patient-Reported Outcome and Quality of Life Instruments Database (PROQOLID): Frequently asked questions

**DOI:** 10.1186/1477-7525-3-12

**Published:** 2005-03-08

**Authors:** Marie-Pierre Emery, Laure-Lou Perrier, Catherine Acquadro

**Affiliations:** 1 Mapi Research Trust, 27 rue de la Villette, 69003 Lyon, France; 2 Mapi Research Institute, 27 rue de la Villette, 69003 Lyon, France

**Keywords:** Patient-Reported Outcomes, Database, Instruments, Health-related Quality of Life, Quality of Life, Questionnaires, Internet, PROQOLID

## Abstract

The exponential development of Patient-Reported Outcomes (PRO) measures in clinical research has led to the creation of the Patient-Reported Outcome and Quality of Life Instruments Database (PROQOLID) to facilitate the selection process of PRO measures in clinical research. The project was initiated by Mapi Research Trust in Lyon, France. Initially called QOLID (Quality of Life Instruments Database), the project's purpose was to provide all those involved in health care evaluation with a comprehensive and unique source of information on PRO and HRQOL measures available through the Internet.

PROQOLID currently describes more than 470 PRO instruments in a structured format. It is available in two levels, non-subscribers and subscribers, at . The first level is free of charge and contains 14 categories of basic useful information on the instruments (e.g. author, objective, original language, list of existing translations, etc.). The second level provides significantly more information about the instruments. It includes review copies of over 350 original instruments, 120 user manuals and 350 translations. Most are available in PDF format. This level is only accessible to annual subscribers. PROQOLID is updated in close collaboration with the instruments' authors on a regular basis. Fifty or more new instruments are added to the database annually.

Today, all of the major pharmaceutical companies, prestigious institutions (such as the FDA, the NIH's National Cancer Institute, the U.S. Veterans Administration), dozens of universities, public institutions and researchers subscribe to PROQOLID on a yearly basis. More than 800 users per day routinely visit the database.

## Review

In clinical research it has become increasingly common to assess the patients' perspective of their symptoms and their impact on their daily life as a tool for determining treatment and a means of evaluating the outcome of the treatment chosen [1,2]. The added value of measuring Patient-Reported Outcomes (PRO) starts to be recognized by key players in the field of clinical research [3]. How patients perceive their health, and the impact of their treatment on their life can provide insight to clinicians previously unavailable [4-6].

However the successful application of PRO studies is dependant on the selection of the appropriate questionnaires for a given application [7,8]. They must be selected according to the domains they measure and the populations and pathologies for which they are designed. Practical issues, such as the availability of different translations, copyrights, and access to instruments are also major criteria in the choice of instruments.

**Figure 1:**
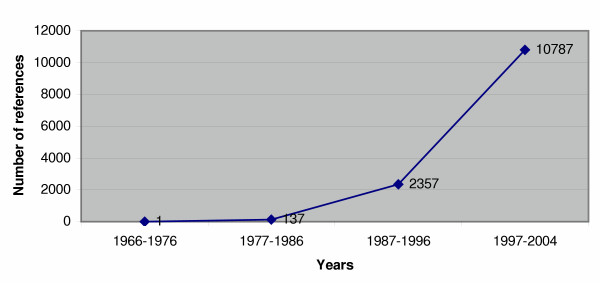
PubMed Search: Number of references with Quality of Life-AND-questionnaire from 1966 to 2004

The search for the most appropriate instruments is hindered by the substantial increase of PRO questionnaires developed in the past ten years. A recent search on PubMed, matching "quality of life" and "questionnaires" shows a striking growth of 450% between the last two decades (Figure 1).

In order to facilitate the selection process the project of a Patient-Reported Outcome and Quality of Life Instruments Database (PROQOLID) was initiated by Mapi Research Trust in Lyon, France. Initially called QOLID (Quality of Life Instruments Database), the project's purpose was to provide all those involved in health care evaluation with a comprehensive and unique source of information on PRO and Health-Related Quality of Life (HRQOL) measures available through the Internet. In collaboration with Dr. Marcello Tamburini (Director, Unit of Psychology, National Cancer Institute, Milan, Italy), the developer of the QLMed.org web site, PROQOLID was launched at the beginning of 2002.

PROQOLID was created by the systematic collection of over 470 validated HRQOL and PRO instruments and their subsequent ordering into categories (e.g. pathologies, conditions, population). Through the structured presentation of synthesized, reliable and constantly updated data on PRO instruments, the PROQOLID database aims to present an overview of existing PRO instruments including all relevant and updated information on each. By providing this information the PROQOLID database facilitates access to the instruments and their developers and eases the process of selecting the instrument appropriate for a given application. Instruments can be chosen through a powerful interactive search function that will allow for the rapid selection of an appropriate instrument. What follows is an overview of the proper usage of the PROQOLID web site through a series of FAQ's collected directly from users of the web site. The PROQOLID database can be accessed on the Internet at .

## Access

### What are the differences in access to PROQOLID's database between members and guests?

The access to PROQOLID is organized in two levels.

#### The guest or free level is available to all visitors at no charge. This level provides brief information for each instrument, including

• Full name of the instrument and acronym

• Author(s)

• Objective

• Pathology

• Disease

• Type of instrument: Coping, Disability/physical functioning, Health status, Psychosocial/psychological, Quality of life, Satisfaction, Social functioning, Symptom/functioning, Utility, Work

• Population: Adolescent, Adult, All, Caregivers, Female, Geriatrics, Male, Pediatrics, Terminal patients

• Mode of administration: Caregiver-administered, Interviewer-administered, Nurse-rated, Physician-rated, Proxy-administered, Self-administered, Telephone-administered

• Number of items

• Original language

• List of existing translations

• Existence of a database: Yes / No

• Time recall

Since 1995, Mapi Research Trust has been a non-profit organization promoting the use and development of Patient Reported Outcomes. In an effort to accomplish this, a significant part of PROQOLID has been made accessible to all users free of charge.

In order to further develop and improve the database and provide additional information on the available instruments, Mapi Research Trust requests a financial participation in order to access PROQOLID'S advanced (or members') level. By subscribing to PROQOLID, you are supporting the continuous collection and update of this unique PRO resource. Membership options are available for pharmaceutical or commercial companies, non-profit organizations, universities, individual academic researchers and students. Benefits to members include a greater degree of practical information on the instruments and, when available, includes the review copy of the instrument, its translations and the user manual, most of them in PDF format.

#### Detailed information of the advanced level available include for each instrument

• Name of the instrument (full and abbreviated)

• Name and contact information of the Authors

• Contact person for information on, or permission to use, the instrument in its original language

• Copyright information

• Detailed conditions of use (e.g. fee, written permission, user agreement etc.)

• Review copy of the original instrument (when possible). Depending on the author's wish, original instruments may be used under specific conditions such as an access fee or signed agreement

• Bibliographic references of the original instrument

• Contact person for information on, or permission to use the translations

• Review copy of the available translations (when possible)

• Bibliographic references of the available translations (when possible)

• Dimensions covered by the instrument

• Time for completion

• Age range

• Scoring: response options, available scores, weighting, score direction and Minimal Important Difference (MID) or Minimal Clinically Important Difference (MCID)

• Existence of a user manual and copy of the user manual (when possible)

• Link to the PRO database identification form, when available

• Methodology of development

• Internal consistency reliability

• Related websites

• Other bibliographic references

## Users

### Who uses PROQOLID?

The web site is available to anyone having an interest in the development, availability and use of Patient-Reported Outcomes (PRO). Through the power of the Internet the PROQOLID project intends to provide this information to the world. Every major pharmaceutical company, non-profit organizations such as the US Food and Drug Administration, the NIH's National Cancer Institute, the Veterans Administration as well as dozens of Universities, researchers and students worldwide subscribe to the advanced level of PROQOLID on a yearly basis. The PROQOLID database is routinely visited by over 800 users per day, thereby educating clinicians, researchers, students, and the world about the availability and proper usage of PRO instruments.

## Content

### How are the instruments organized in the PROQOLID database?

The PROQOLID database was created in an effort to provide a means to facilitate the search process for and provide more efficient searches of any given PRO instrument. By organizing instruments in the PROQOLID database by several easy to understand categories, both time and energy are saved by the user. The different categories can be located on the Search page of the web site or by accessing directly from the tool bar at the top of the page. The different categories are as follows:

#### Alphabetical

The purpose of the Alphabetical list is to provide an overview of all existing PRO instruments. Over 1000 instruments are listed in alphabetical order according to their abbreviated name (or acronym). Some of the instruments are only listed, and these are displayed in standard font. For the remaining instruments access is available by simply clicking on the green link containing the abbreviated name of the instrument. Instruments can be accessed through an interactive letterbox at the top of the page. For example if the instrument begins with "D", simply click on the "D" at the top of the page and all instruments beginning with that letter will be displayed alphabetically.

#### Generic Instruments

The generic instruments are listed by alphabetic order on a separate web page.

#### Pathology/disease

A specific web page is dedicated to each pathology, and the instruments are listed either as generic instruments of the pathology or as disease-specific. The classification is structured based on Medline's Medical Subject Headings (MeSH) to ensure that the concepts are widely accepted. Please note that some diseases may be part of several pathologies. For example the disease "dementia" is part of both the pathologies "Neurology" and "Psychiatry/Psychology".

#### Population

The web page lists the instruments as they apply to specific populations including Adolescent, Adult, All, Caregivers, Female, Geriatrics, Male, Pediatrics, and Terminal patients.

#### Author's name

Instruments are grouped alphabetically according to the author's name, and as in the alphabetical list a letterbox is provided at the top of the page.

#### Search engine

You may search for instruments according to 10 criteria, including the name of the instrument, the pathology, the population or the available languages. The various criteria may be crossed referenced using the following Boolean Operators: AND, OR, NOT.

### How many instruments are contained in PROQOLID?

The PROQOLID database was developed and is updated in close collaboration with the instruments' developers. It currently describes over 470 PRO instruments according to a structured format. The list is currently growing at the rate of fifty instruments per year. The database also includes review copies of 350 original instruments and 350 translations, most of them in PDF format. In order to determine the available translations simply access the instrument on the web site and all existing translations are conveniently listed. Also available through PROQOLID are over 125 associated User Manuals, and the description of 80 separate PRO databases. A fifth update of the whole database is underway and will include new information for each questionnaire on the reproducibility (or test-retest reliability) and clinical validity. In addition the PROQOLID website contains links to 150 external Internet resources relevant to the field of PRO usage and development. A general question and answer section on PRO is also included as well as on line full text articles on the development, validation, and linguistic adaptation of many of the PRO instruments published in medical journals.

### What are the instruments criteria to be eligible for inclusion in PROQOLID?

To be eligible for inclusion in the database an instrument must be the subject of a publication that describes its development and/or validation. There is no charge to authors who wish to insert their instruments in PROQOLID nor are authors paid for their participation in this program. The Mapi Research Trust in Lyon, France determines ultimate decision for inclusion.

## Search

### How does the Search Engine work?

Besides the search by categories of instruments listed above (i.e. alphabetical list, generic, pathology/disease, population and author's name), an interactive search engine is included in the PROQOLID web site. All of the instruments contained on the web site can be accessed from this location. Searches can be made by:

• Abbreviated Name

• Full Name

• Author

• Pathology

• Disease

• Type of Instrument

• Population

• Mode of Administration

• Inclusion of a PRO Database

• Language

In an effort to increase the effectiveness of each search the ability exists to include up to nine (9) sub-parameters per search with the stipulation of "AND" or "OR". For example one could enter Author "=" Anderson J "OR" Author "=" Anderson R "AND" Type of Instrument "=" Quality of Life, "AND" Language "=" French, or any other combination that would suit the users needs. Through this function a user is able to drastically narrow the number of instruments displayed in the results window, thereby saving time and effort. If questions exist on the functioning of the search engine, or any questions about the PROQOLID database a contact page is provided with names and e-mail addresses for Mapi Research Trust and individual site managers in both Europe and North America. Additionally a video including a demo of PROQOLID can be seen on the home page.

## Update

### Who maintains the PROQOLID database?

Mapi Research Trust has maintained the PROQOLID database for three years. The information contained in the website for each instrument is updated at least once a year in collaboration with the instruments' developers and over fifty new instruments are added to the website each year. Mapi Research Trust has listened to the needs of the Pharmaceutical Industry, Industry Regulators, health care professionals, and patients. With the passing of time the organisation has developed into an intricate team of professionals whose single goal is to define and unite the various requirements of each of these groups in order to provide better communication and understanding of each groups needs. PROQOLID achieves to translate these objectives into a concrete application which ultimate goal is the improvement of the patients' quality of life and health outcomes.

## Conclusion

The Patient-Reported Outcome and Quality of Life Instruments (PROQOLID) database aims to present an overview of existing PRO instruments. PROQOLID currently describes more than 470 PRO instruments in a structured format. It includes review copies of over 350 original instruments, 120 user manuals and 350 translations. Most are available in PDF format. The database is updated in close collaboration with the instruments' authors on a regular basis. Fifty or more new instruments are added annually. By providing this information the PROQOLID database facilitates access to the instruments and their developers and eases the process of selecting the most appropriate instrument for a given application. Instruments can be chosen through a powerful interactive search function. The PROQOLID database can be accessed on the Internet at .

## Authors' contributions

MPE conceived the database and participated in its implementation and helped to draft the manuscript. CA helped in the conception of the database and drafted the manuscript. LLP has contributed in the design, coordination and follow up of the database. All authors read and approved the final manuscript.
